# *Clostridium difficile* Infection: Epidemiology, Pathogenesis, Risk Factors, and Therapeutic Options

**DOI:** 10.1155/2014/916826

**Published:** 2014-06-01

**Authors:** Mehdi Goudarzi, Sima Sadat Seyedjavadi, Hossein Goudarzi, Elnaz Mehdizadeh Aghdam, Saeed Nazeri

**Affiliations:** ^1^Department of Microbiology, School of Medicine, Shahid Beheshti University of Medical Science, Tehran, Iran; ^2^Department of Pharmaceutical Biotechnology, Pasteur Institute of Iran (IPI), No. 358, 12th Farwardin Avenue, Jomhhoori Street, Tehran 1316943551, Iran; ^3^Department of Pharmaceutical Biotechnology, Faculty of Pharmacy, Tabriz University of Medical Sciences, Tabriz, Iran

## Abstract

The incidence and mortality rate of *Clostridium difficile* infection have increased remarkably in both hospital and community settings during the last two decades. The growth of infection may be caused by multiple factors including inappropriate antibiotic usage, poor standards of environmental cleanliness, changes in infection control practices, large outbreaks of *C. difficile* infection in hospitals, alteration of circulating strains of *C. difficile*, and spread of hypervirulent strains. Detection of high-risk populations could be helpful for prompt diagnosis and consequent treatment of patients suffering from *C. difficile* infection. Metronidazole and oral vancomycin are recommended antibiotics for the treatment of initial infection. Current treatments for *C. difficile* infection consist of supportive care, discontinuing the unnecessary antibiotic, and specific antimicrobial therapy. Moreover, novel approaches include fidaxomicin therapy, monoclonal antibodies, and fecal microbiota transplantation mediated therapy. Fecal microbiota transplantation has shown relevant efficacy to overcome *C. difficile* infection and reduce its recurrence.

## 1. Introduction

The name “*Clostridium difficile*” (*C. difficile*) comes from the Greek word “Kloster” meaning spindle. At first, due to the isolation difficulty and the requirement of anaerobic culture condition, the bacterium was given the name “*Bacillus difficilis*” in 1935 [1]. It later became clear that this microorganism is able to produce toxins and the name was subsequently changed to* C. difficile* in the 1970s [2]. The pathogenicity associated with* C. difficile* was first described in germ-free rats in 1969 [3]. In 1893, the first description of pseudomembranous colitis (PMC) was reported and, in 1974, the association between receiving clindamycin and PMC patients was reported [4].

*C. difficile* is gram-positive rod, spore forming, strict anaerobic bacillus and is part of the normal intestinal microbiota in 1–3% of healthy adults and 15–20% of infants. The mentioned statistics would be increased considerably during long hospitalization and after surgery.

The important disorder caused by this bacterium is often termed “*C. difficile*-associated diarrhea” or* C. difficile* infection (CDI). CDI is one of the most prevalent problems in hospitals and nursing homes where patients frequently receive antibiotics [5].

## 2. Epidemiology

In the last two decades, the incidence and the mortality rate of CDI have considerably increased substantially in both hospital and community settings due to the spread of hypervirulent strains and improper administration of antibiotics [6]. The epidemiology of CDI in North America, Europe, and some part of Asia is well documented [7]. Recent epidemiological reports from the United States implied that* C. difficile* has replaced methicillin-resistant* Staphylococcus aureus* as the most common cause of the healthcare-associated infection [8]. Based on the several reports from US, Canada, and Europe, the incidence of CDI has increased by 2- to 4-fold in the past decade, particularly in the elder patients with the exposure to the health care settings such as long-term care facilities and hospitals. For instance, Québec experienced a large outbreak of CDI and noted a 4-fold increase in CDI between 1998 and 2004, with overall mortality of 6.9% [9]. The European Study Group of* C. difficile* (ESGCD) reported the mean incidence of healthcare-associated CDI as 4.1 per 10000 hospital patient days [10]. The incidence of community-acquired* C. difficile* infection (CA-CDI) is also increasing in the community settings. Consequently, different studies performed in US, Canada, and Europe suggested that approximately 20%–27% of all CDI cases were community associated, with the mean incidence of 20–30 per 100000 populations [11]. Approximately 11–28% CDI infection is acquired in the community, which seems to be consistent in different countries. More recently, US studies have reported that the incidence rates of CA-CDI varied between 6.9 and 46 cases per 100000 person-years.

Children and peripartum women populations previously described as the low risk for CDI show the increased incidence now [12]. Annual rates of pediatric CDI-related hospitalizations in US increased from 7.24 per 10000 hospitalizations in 1997 to 12.8 in 2006. In a study conducted in 4 states of US in 2005, severe cases of CDI in peripartum women were reported. Additionally, the rates of US hospital discharges of peripartum women showed that the CDI increases significantly between 2004 and 2006, from 0.04 to 0.07 per 1000 discharges [13]. The rate elevation of the incidence, severity, mortality, and recurrence of CDI have been attributed largely to the spread of a new strain of* C. difficile*, designated North American pulsed-field gel electrophoresis type 1 (NAP1), polymerase chain reaction (PCR) ribotype 027, toxinotype III, and restriction endonuclease analysis type BI (i.e., BI/NAP1/027). Ribotype 027 strains were first reported in Canada in 2003 and shortly thereafter in the UK. NAP1/027/BI strain is associated with its ability to produce high concentrations of toxins, high transmissibility, high sporulation, production of binary toxins, high level of resistance to fluoroquinolone due to the mutations in gyrA, and variation in the tcdC repressor gene (which could result in the increased toxin A (16-fold) and toxin B (23-fold)). Moreover, the polymorphisms in tcdB could result in improved toxin binding. There are conflicting reports regarding the severity of disease induced by 027/NAP1 in comparison to disease severity caused by other strains. This strain isolated from most US and Europe area has variable distributions among different countries. Other emerging hypervirulent genotypes may present an equivalent threat in terms of disease severity [14].

The molecular epidemiology of* C. difficile* is varied; a different ribotype can predominate in a particular area during certain periods and at the same time is extremely rare elsewhere. For example, in a study conducted on 894* C. difficile* isolates from patients enrolled from 16 countries on three continents, it was shown that ribotype 027 strains were the most common strains identified and were widely distributed throughout North America but restricted to three of thirteen countries in Europe. Ribotype 001 isolates were the most common strains identified in Europe [15].

Despite the widespread existence of hypervirulent epidemic strains 027, 001, and 078 in Europe and North America, sporadic cases of CDI caused by the 027 strain were recently reported from the hospitals in Japan, Korea, Hong Kong, and Australia. However, they do not seem to be established in Asia [16, 17]. In a study conducted by Collins et al. in order to better understand the epidemiology of CDI in Asia it became clear that ribotypes smz/018 and 017 were dominant ribotypes that lead to epidemic infections. The widespread prevalence of the 017 group of A-B+ strains in Asian countries exhibits that laboratory methods for toxin B are preferable to toxin A assays in order to diagnose CDI [17]. Other genotypes of* C. difficile* have been also shown to be predominant or associated with the infection outbreaks or severe cases. For example, PCR ribotypes 053 in Austria, 106 in United Kingdom, 001 in China and Korea, and 002 and 014 in Japan are predominant ribotypes [16–18].

## 3. Pathogenesis

Infections of* C. difficile* can be categorized as endogenous or exogenous. Endogenous infection originates via the carrier strains whereas exogenous infection occurs through infected individuals, contaminated health care workers, nosocomial sources, and contaminated environment [19].* C. difficile* is spread via the oral-fecal route. It is acquired by oral ingestion of spores which are resistant in the environment as well as being tolerant of the acidity of the stomach. In the small intestine, ingested spores are germinated to the vegetative form. Besides, due to the application of antimicrobial agents and disruption of the normal colonic bacteria, colonization of the* C. difficile* occurs in the large intestine. Subsequently, bacterial growth, multiplication, and toxin production damage entrecotes in the intestinal crypts [4–6].

The primary produced toxins by this bacterium are toxins A (an enterotoxin) and B (a cytotoxin). Although the evidence has suggested toxin A as the major toxin, toxin B producing* C. difficile* strains causes the same spectrum of diseases as strains which produce both toxins. Besides, toxin A (TcdA) and toxin B (TcdB) are the major virulence factors of* C. difficile* contributed to its pathogenicity which induces mucosal inflammation and diarrhea [20]. In addition to the major toxins,* C. difficile* may produce a number of other putative virulence factors, including CDT binary toxin, fibronectin binding protein FbpA, fimbriae, SlpA S-layer, Cwp84 cysteine protease, and Cwp66 and CwpV adhesions [20].

## 4. Risk Factors

Recognition of high-risk populations is helpful for prompt diagnosis and treatment of patients with CDI. The categorized risk factors for developing CDI usually include primary risk factors and secondary risk factors [21].

The most important primary risk factors include male gender, age more than 65 years, age less than 1 year with comorbidity or underlying conditions, prolonged duration of hospital stay, and antimicrobial therapy. The most important secondary risk factors include comorbidity or underlying conditions, inflammatory bowel diseases (IBDs), immunodeficiency and HIV, malnutrition, low serum albumin level (<2.5 g/dL), neoplastic diseases, cystic fibrosis, and diabetes [22]. Administration of broad-spectrum antimicrobials that impair the growth of normal flora and promote proliferation of toxigenic* C. difficile* remains the most widely recognized risk factor. Therefore, antimicrobial therapy plays a central role in the development of CDI. Any kind of antibiotics mainly clindamycin, cephalosporins, fluoroquinolones (moxifloxacin, gatifloxacin, and levofloxacin), ampicillin/amoxicillin, macrolides, co-trimoxazole, and tetracyclines can cause CDI. The exposure to metronidazole and vancomycin, which are used as the first choice drugs for treatment of CDI, may result in CDI themselves [21, 22].

Cancer chemotherapy drugs possessing antimicrobial activity may also be associated with the increased risk of CDI. Conflicting results have been published on the role of proton pump inhibitors (PPIs) and H2 blockers in the development of CDI. They appear to be much less important than antibiotics [21–23].

Although many factors are involved in CA-CDI, according to the several studies, consumption of contaminated meat and food is an important risk factor for CA-CDI [23].

## 5. Clinical Presentations

*C. difficile* is an important nosocomial pathogen and the most frequently diagnosed cause of infectious diarrhea in the hospitalized patients. Hospital-acquired CDI (HA-CDI) defined as the onset of symptoms occurs more than 48 hours after admission to the health care facility or less than 4 weeks after being discharged. However, a substantial percentage of CDIs occur in individuals who neither received antibiotic therapy nor were hospitalized recently. The mentioned group was recognized as the community-acquired CDI defined as symptom onset in the community or during the first 48 hours after admission to the hospital, in the case of no hospitalization in the past 12 weeks. The onset of symptoms occurring in the community between 4 and 12 weeks after discharge from the hospital is defined as indistinctive CDI [24].

### 5.1. Carrier Stage

Carriers are individuals who shed* C. difficile* in their stools but do not have diarrhea and depending on their status may be as the reservoirs of* C. difficile*. According to several studies, the frequency of carrier stage in the healthy adults, hospitalized patients, and patients with long hospital stays is approximately 3%, 20–30%, and 50%, respectively. Reportedly, the asymptomatic patients infected with clostridium are served as potential reservoirs for continued* C. difficile* contamination of the hospital environment. Consequently, the carriers facilitate the spread of the spores into the environment at lower concentrations than patients with diarrhea or other symptoms [25].

### 5.2. *C. difficile*-Associated Diarrhea (CDAD)

*C. difficile* is the cause of approximately 25–30% of all cases of antibiotic-associated diarrhea (AAD). It is defined as unexplained diarrhea occurring between 2 hours to 2 months after use of antibiotics and often accompanied by abdominal pain and cramps [24, 25]. Diarrhea was defined as the passage of 3 or more unformed stools for at least 2 consecutive days. Besides, CDAD is established when toxin A is identified in stool, regardless of* C. difficile* isolation from stool. In the past CDAD almost was thought to be related to hospitalization. However, according to Centers for Disease Control (CDC) reports in recent years, exposure is the most important risk factor for CDAD [26].

Although literature review shows that different groups of antibiotics are associated with CDAD in hospitalized patients, the important related antibiotic or antibiotic group is still not clear. However, there are two hypotheses about acquisition and pathogenesis of CDAD. In the first hypothesis, a patient acquires* C. difficile* during hospitalization and is subsequently at risk of CDAD when exposed to antimicrobial agents. In the other hypothesis a patient acquires* C. difficile* during hospitalization but is not highly susceptible to* C. difficile* infection until receiving antimicrobial therapy [24, 26].

### 5.3. *C. difficile*-Associated Colitis (CDAC)

Colitis without pseudomembrane formation is the most common clinical manifestation of CDI. CDAC results in significant healthcare costs, prolonged hospitalizations, and increased morbidity. The symptoms are including abdominal pain, nausea, malaise, anorexia, watery diarrhea, and possible presence of trace blood in the stool. In addition, low grade fever, dehydration, pyrexia, and leukocytosis may occur. High white blood cell count (WBC) must be considered carefully for CDI in the patients treated with antibacterial agents, even in the absence of diarrhea [19].

### 5.4. Pseudomembranous Colitis (PMC)

PMC is a descriptive term for the form of colitis that first was described as the postoperative complication of gastrojejunostomy for an obstructive peptic ulcer [19]. In recent years, the majority of pseudomembranous colitis cases have been ascribed to the antimicrobial treatment which altered patient's normal flora. Approximately, the majority of PMC cases are related to the use of clindamycin and lincomycin. However, a number of other related antibacterial agents have been reported [27].

Clinical manifestations of PMC are including abdominal cramp, dehydration, hypoalbuminemia (less than 30 mg/L), watery diarrhea, and rising of inflammatory cells, serum proteins, and mucus. Furthermore, 2–10 mm yellowish plaques are observed in colorectal mucosa and sometimes in the terminal ileum following sigmoidoscopic examination and are the best detection signs of PMC. Because of the potential toxic effects of the infection, it is essential to select the appropriate antibacterial agents for treatment of pseudomembranous colitis. It should be noted that relapses occur in about 10–25% of cured patients [19, 28].

### 5.5. Fulminant Colitis

Fulminant colitis, which occurs approximately in 3% of CDI patients, accounts for most of the serious complications including perforation, prolonged ileus, megacolon, and death. A significant rise of fulminant colitis in recent years is associated with a hypervirulent strain of* C. difficile* which results in the development of symptoms, multiple organ failure, and increased mortality [29].

Besides, several studies have reported the importance of* C. difficile* infection in inflammatory bowel disease (IBD). IBD could represent a clinical challenge because of some symptom similarity with CDI, even in the absence of recent antibiotic administration.* C. difficile* has also been reported to be involved in the exacerbation of ulcerative colitis (UC). It is necessary to routinely evaluate the* C. difficile* in patients with severe IBD especially before initiating further immunosuppressive therapy. However, detection of* C. difficile* in patients suffering UC is so difficult because of the wide spectrum of diseases [29, 30].

### 5.6. Recurrent CDI

Recurrent CDI is one of the greatest challenging aspects of CDI that occurs either due to relapse or reinfection. The relative frequency of each mechanism of recurrence has not been well described; however, in many of published articles, 33%–75% of cases of recurrent CDI are attributed to the infection with a new strain [10]. Approximately 25% of patients treated with metronidazole or vancomycin, typically within 4 weeks of completing antibiotic therapy, experience recurrent symptoms. The main cause of recurrent CDI has not been recognized, but it seems that disturbance of the normal bowel flora and defective immune response against* C. difficile* and/or its toxins play the important role in the development of recurrent CDI [10, 23].

### 5.7. Extracolonic Infections

Recent studies demonstrated that CDI is not only limited to the colon. In fact, extracolonic* C. difficile* infections have been reported and clinical manifestation of disease includes small bowel disease with formation of pseudomembranes on ileal mucosa, bacteremia, reactive arthritis, visceral abscess, appendicitis, intraabdominal abscess, osteomyelitis, and empyema. In most cases, extracolonic* C. difficile* infections have previous involvement with underlying diseases such as gastrointestinal diseases, either* C. difficile* colitis or surgical and anatomical disruption of the colon [5, 19].

## 6. Diagnosis

According to the clinical criteria, the diagnosis of* C. difficile* is based on the appropriate clinical context, history of recent antibiotic administration, and diarrhea. Other signs such as fever, abdominal pain, leukocytosis, and pyrexia in combination with laboratory testing are suggested for the diagnosis [27]. Recently, the Society for Healthcare Epidemiology of America (SHEA) and the Infectious Diseases Society of America (IDSA) published the guidelines for the management of patients with CDI. According to the SHEA/IDSA guidelines, all of the laboratory tests should be done on the unformed stool specimens unless ileus is suspected [31]. Owing to the presence of both toxigenic and nontoxigenic strains of* C. difficile* in asymptomatic patients, testing is not necessary for infected or cured people. In addition, processing a single specimen from symptomatic patient usually is sufficient and routine testing of multiple specimens is not recommended by SHEA and IDSA. Moreover, repeated testing during the same episode of diarrhea is of limited value. There are many different diagnostic tests for the identification of* C. difficile* infection. It is important to be aware of the limitations of each test and the need to follow protocols for proper sample selection and handling [31, 32].

### 6.1. Diagnostic Tests

#### 6.1.1. Laboratory Diagnostic Tests

*Transport and Storage of Samples.* Watery diarrhea or loose stools are the best specimen for the diagnosis of CDAD. Faecal samples should be as fresh as possible and submitted in a clean, watertight container. Enhancing the recovery of* C. difficile* and its toxins by transport media or anaerobic condition has not been recommended due to the raising of false positive rate. Specimens should be transported immediately and stored at 2° to 8°C until being tested because of toxin inactivation in room temperature [33]. Moreover, repeated freezing and thawing of the specimen should be avoided for the same reason. Phosphate buffer solution (PBS) may be useful for preservation of* C. difficile* viability in the transport and storage state. For long-time storage, faecal samples should be saved in PBS at 4°C. For outbreak investigation, it is recommended to store toxin-positive samples at 4 or −20°C. One or two specimens from patient with diarrhea are sufficient for detection of* C. difficile*. Testing three stools can increase the likelihood of a positive test by 10% [33, 34].

*Culture.* In the clinical laboratories, the high cost and the need for anaerobic facilities and expert technicians make the* C. difficile* culturing so demanding which is not routinely performed. As a result, it is recommended to culture the bacterium in the case of consultation with infectious disease and/or gastroenterology specialists [33, 35]. Cycloserine-cefoxitin-fructose agar (CCFA), as a selective and differential agar medium, is the first choice of isolation media for the recovery of* C. difficile* from fecal specimens. Cultured isolates are important for epidemiological investigations. Once an organism has been recovered, it is necessary to perform a toxin test to confirm the ability of toxin production [32, 35].

*Toxin Assay. C. difficile* toxins can be detected by several methods as mentioned below.

*(1) Cell Culture Neutralization Assay (CCNA)*. CCNA is a high sensitive and specific test based on the detection of* C. difficile* toxin B in cell culture. It is more sensitive than toxin detection by immunoassays [19]. However, it is time-consuming and labor-intensive and requires special laboratory facilities. Employed cell lines in this method are Vero, Hep-2, ovary, heLa, MRC-5 lung fibroblast, and Chinese hamster. Cell cytotoxicity tests showed the sensitivity of 57–100% and the specificity of 99-100% in different studies [19, 32].

*(2) Immunoassay.* Immunoassay tests are available for detection of toxin A alone or both toxins A and B. Two main types of immunoassay methods are enzyme immunoassay (EIA) and immunochromatography. EIA methods are easier, faster to perform, and less expensive than CCNA and have a sensitivity of 75% to 95% and a specificity of 83% to 98% in comparison to CCNA. Enzyme linked immunosorbent assay (ELISA), as a technique based on the EIA, is able to detect toxin A alone or both toxins. ELISA shows the sensitivity and the specificity of 50–90% and 70–95%, respectively [32, 36]. In the case of EIAs and CCNAs insensitivity to toxin A/B, testing algorithms using a mitochondrial glutamate dehydrogenase (GDH) assay is applied by several laboratories as an initial screening marker for the presence of* C. difficile* in stool samples. GDH testing has been reported to have sensitivities from 75% to more than 90%, with negative predictive values of 95% to 100% in the appropriate clinical setting. Moreover, dot immunobinding, immunochromatography assay, and monoclonal antibody against toxins are the other immunoassay tests for detection of* C. difficile* toxins [33, 36, 37].

*Nucleic Acid Amplification Methods. *Nucleic acid amplification tests (NAATs) are the most recent methods for detection of* C. difficile*. Available NAATs to identify genes of* C. difficile* are PCR, real-time PCR, and loop-mediated isothermal amplification (LAMP) [38]. These tests detect various targets within the pathogenicity locus of the* C. difficile* genome such as tcdA, tcdB, tcdC, and the other genes such as 16S,* glu*D, and triose phosphate isomerase (TPI)* C. difficile* housekeeping gene [39]. PCR assays as potential replacements for the less-sensitive (EIA) and less-specific (GDH) assays have the sensitivity and specificity of 90%–100% and 94%–100%, respectively [32, 35].

*Latex Agglutination Assay. *Latex agglutination assay, which detects glutamate dehydrogenase, is a rapid, relatively inexpensive, and specific test. However, it would not be used as a routine laboratory procedure for identification of* C. difficile* [19]. 

*Other Tests. *Methods such as gram staining, counterimmunoelectrophoresis, chromatography, rapid membrane tests, and analysis of fecal leucocytes and blood compared to other assays demonstrate low sensitivity and specificity [19, 33, 40].

#### 6.1.2. Nonlaboratory Based Tests

Endoscopy (sigmoidoscopy and colonoscopy) is an invasive test that is generally not employed to do an initial diagnosis of CDI unless there is a high level of suspicion regardless of normal stool tests results. In patients with PMC, detection is based on the direct visualization using either sigmoidoscopy or colonoscopy [27]. Although endoscopy is required for the specific diagnosis of PMC, it is not sufficient to diagnose all the cases of CDAD. Colonoscopy in patients with fulminate colitis raises the risk of bowel perforation. Computed tomography (CT) scan, as a noninvasive method with low sensitivity and specificity, is uncommonly used to make the initial diagnosis of PMC or fulminant CDI. It may be helpful in the assessing of disease severity and determining the presence of perforation [22, 27, 41].

## 7. Treatment

Treatment of CDI is not recommended in asymptomatic individuals since available data suggest that treatment of asymptomatic individuals would not prevent symptomatic transmission or infection. Various treatments are taken based on the severity of the patient's illness and whether one is treating initial infection or recurrent CDI. Treatment in cases of CDI is classified in two main categories, nonsurgical and surgical treatments [31, 41].

### 7.1. Nonsurgical Treatment

Short period antibiotic therapy is clinically effective for the small percentages of patients, but specific antimicrobial therapy is necessary in the majority of patients. The use of antimotility agents such as narcotics and loperamide is not recommended because they may increase the severity of colitis. Empiric antibiotic therapy in patients with severe diarrhea and at risk population should start immediately while stool test results are pending [41, 42].

Metronidazole and oral vancomycin are recommended as antibiotics for the treatment of initial episode. Metronidazole as an inexpensive and effective first-line drug with low level of resistance and few adverse effects is employed for the treatment of mild to moderate disease in either oral or intravenous route but should not be used for critically ill patients [22]. Metronidazole has similar efficacy as vancomycin for treatment of mild to moderate CDI, but it is not approved by the US Food and Drug Administration (FDA) for treatment of CDI. Unlike vancomycin, metronidazole has well absorption and its fecal concentration is very low or none in the healthy volunteers and asymptomatic* C. difficile* carriage [22, 42].

At a dosage of 500 mg orally 3 times a day or 250 mg orally, given 4 times a day for 10 days, metronidazole is first line for the mild to moderate CDI. Oral vancomycin, 500 mg 4 times daily for 10 days, is administered in the patients who cannot tolerate metronidazole. Administration of vancomycin via enema is used for patients with surgical or anatomic abnormalities. Importantly, the routine use of vancomycin is not recommended due to the risk of development of vancomycin resistantance in other organisms especially enterococci [22, 42, 43]. However, in the case of severe CDI, treatment with oral vancomycin is recommended. On the other hand, in the case of treatment failure with low dose of oral vancomycin and also patients with complicated CDI, it is recommended to use high-dose (250–500 mg every 6 hours) oral vancomycin plus intravenous metronidazole, 500 mg 3 times a day [31, 42]. 15–50% relapse rate may occur after vancomycin treatment. As mentioned, approximately 15% and 20% of treated CDI patients will experience a recurrence of disease within 4 weeks after the treatment [23, 41, 42]. Treatment of the first recurrence of CDI is the same as the treatment of first episode of CDI. In patients with a second recurrence of CDI, vancomycin should be the treatment of choice. Tapered or pulse-dosage vancomycin may reduce the risk of a subsequent recurrence [44].

Fidaxomicin is a new macrocyclic that might be favored over the oral vancomycin in patients with multiple recurrences. The low rate of antibiotic resistance and the minimal effect on the fecal microbiota and preventing relapses caused FDA to approve fidaxomicin for treatment of CDI [45]. Fidaxomicin can be applied for treatment of patients at high risk of recurrent CDI, patients infected with the nonhypervirulent strain, patients with multiple episodes of recurrence, and patients who are not able to tolerate oral vancomycin [41, 42]. Other antibiotics which may be used against* C. difficile* include fusidic acid, teicoplanin, rifaximin, ramoplanin, nitazoxanide, and tigecycline.

Several therapeutic protocols can be employed for patients with a third or subsequent recurrence of CDI including the following options: oral vancomycin, 125 mg 4 times a day for 14 days, followed by rifaximin, 400 mg twice daily for 14 days, or intravenous immunoglobulin, 400 mg/kg, repeated up to 3 times at 3-week intervals, or combination therapy with oral vancomycin, oral rifaximin, and fecal microbiota transplantation (FMT) [22].

FMT is an alternative therapy for treatment of recurrent cases of CDI. In this method, normal fecal microbiota in patients is restored using intestinal microorganisms from a healthy donor stool. To date, several studies showed the high success rate of FMT treating CDI with rapid and enduring response [46]. Gough et al. reported the effectiveness of 92% of cases in the case of fecal transplant as an alternative treatment of recurrent CDI. In other studies, the success rate of FMT via enema, nasogastric route, and colonoscopy was 95%, 76%, and 89%, respectively [47].

### 7.2. Surgical Treatment

Surgery is a therapeutic option for treatment of fulminant colitis or those patients who are not responding to medical therapy. In patients who do not respond to optimal medical therapy or have symptoms of megacolon or sepsis, it is therefore recommended to do a surgical consultation earlier. In early fulminant colitis cases, any delay in the surgery can result in death [48]. CT of the abdomen may provide valuable data in assessing disease severity and the need for surgical intervention [27, 41].

## 8. Prevention

Effort on the prevention of initial CDI, especially in health care settings, is indispensable. The bases of these efforts are reduction of the prolonged use of multiple antibiotics and prevention of transmission from patient to patient.

### 8.1. Antimicrobial Stewardship

Appropriate and accurate use of one single antimicrobial in patients at high risk of CDI and improvement of overall prescribing practices are two important approaches to development of antimicrobial stewardship. There is also a joint IDSA/SHEA guideline on establishing an institutional program to enhance antimicrobial stewardship [22, 31, 41, 42].

### 8.2. Reduction of Transmission

*C. difficile* is transmitted via spores picked up either by indirect contact with a contaminated surface or by direct contact with an infected person (people in the hospital, presumably via their hands). In recent years, it has been established that contamination of surfaces and equipment plays a critical role in the* C. difficile* infection transmission between patients. Spore form of* C. difficile* is considered as a vehicle for the transmission of CDI [49]. It is postulated that some of the strains including 027 and 001 show higher ability to sporulate than other strains. Applying sodium hypochlorite, chlorine dioxide products, and chlorine solutions has been demonstrated to be effective in killing* C. difficile* spores. However, alcohols, chlorhexidine, hexachlorophene, and many disinfectant agents employed routinely in antiseptic hand wash or cleansers have been exhibited to be ineffective against* C. difficile* spores [10, 33, 49]. Infectious Diseases Society of America (IDSA)/Society for Healthcare Epidemiology of America (SHEA) has recommended hypochlorite solutions (5,000 ppm) to inhibit continuous transmission via environmental disinfection in outbreak settings and reduction of environmental contamination in the areas with increased rates of CDI [31].

Hands of healthcare workers (HCWs) are one of the important routes of* C. difficile* transmission. It is necessary that HCWs wash their hands with soap and water to mechanically remove spores from the hands [10, 50]. Although many of the challenging studies believed that handwashing with soap and water (or an antiseptic soap) is more effective than waterless alcohol-based hand rubs for removing* C. difficile*, the use of alcohol-based hand rubs is still an effective way to reduce the overall incidence of health care-associated infections [51].

Major points to reduce the transmission include

1. contact precaution example, for example, wearing gloves, aprons, or gowns when caring for the patient;
2. appropriate hand hygiene;
3. use of sporicidal agents for environmental cleaning and disinfection.

Moreover, new technologies for room disinfection have been investigated including “no-touch” methods (room disinfection by using ultraviolet (UV) light or gaseous hydrogen peroxide) and self-disinfecting surfaces (copper coating of room surfaces) [10].

### 8.3. Probiotics

Probiotics are live microorganisms that confer and/or improve a health benefit to the host via the following ways: enhancing immunity, reestablishing the balance of intestinal flora, and protecting intestinal barrier. Although they are used as preventive and therapeutic agents, their role in the treatment and prevention of CDI remains controversial [52]. The best studied probiotic agents in CDI are* Saccharomyces boulardii* and* Lactobacillus*. Several studies showed that the mixtures of probiotics can be useful in the treatment and prevention of ADD and CDI [53].

### 8.4. Vaccine

One of the approaches to prevention of* C. difficile* infection is the development of an effective vaccine. Toxoids A and B are the best candidates for* C. difficile* vaccine and they are able to exert excellent serum antibody responses in healthy adults [54]. As a first report of a DNA vaccine targeting* C. difficile* toxins, Gardiner et al. explained the receptor-binding domain of* C. difficile* toxin A that is able to induce well immune responses in mice and protect them from death. The* C. difficile* vaccine must be further studied in the clinical trials [55].

## 9. Conclusion

CDI is a serious problem in the healthcare with an increasing incidence worldwide which can cause significant morbidity and mortality. Considering the increases of CDI incidence even in the populations previously thought to be at lowrisk and also in order to identify populations at risk, monitor the incidence, and characterize the molecular epidemiology of strains, it is essential that healthcare facilities and scientific societies revisit their national surveillance for infection control. Recurrent CDI as a major management challenge not only is difficult to treat but also may affect patients for a long time. Obviously, treatments currently available for CDI are inadequate. New options for treatment of CDI are including novel antibiotics (e.g., fidaxomicin), fecal microbiota transplant, vaccines, monoclonal antibodies, and probiotic therapy (employing* S. boulardii*). Appropriate use of antibiotics and contact precautions, for example, using gloves, hand washing, and environmental disinfection, along with integrated surveillance programs can be effective for the control of CDI outbreaks.
